# Supplementary material for: Long-term changes in the Juvenile Sockeye Salmon Rearing Capacity of the Chignik Lakes Watershed
Source: PLoS One. 2026 May 19;21(5):e0349239. doi: 10.1371/journal.pone.0349239 (PMC13186349; doi:10.1371/journal.pone.0349239)
Supplement: S1 Table — (DOCX) [file pone.0349239.s001.docx]

**S1 Table. Linear regression statistics for mean monthly surface water temperatures.**

Ordinary least squares regressions of mean monthly surface water temperature fit separately for Black Lake and Chignik Lake in each of June, July, and August from 1990–2023.

| Lake | Month | *n*Years | Slope (°C yr^−1^) | SE | *t* | df | *p* | *R*^2^ |
| --- | --- | --- | --- | --- | --- | --- | --- | --- |
| Black | June | 27 | 0.0280 | 0.0276 | 1.01 | 24 | 0.321 | 0.041 |
| Black | July | 25 | 0.0191 | 0.0281 | 0.68 | 23 | 0.504 | 0.020 |
| Black | Aug | 21 | 0.0306 | 0.0406 | 0.75 | 19 | 0.460 | 0.029 |
| Chignik | June | 31 | 0.0140 | 0.0259 | 0.54 | 29 | 0.592 | 0.010 |
| Chignik | July | 26 | 0.0150 | 0.0239 | 0.63 | 24 | 0.538 | 0.016 |
| Chignik | Aug | 21 | 0.0318 | 0.0241 | 1.32 | 19 | 0.203 | 0.084 |
