# Supplementary material for: Long-term changes in the Juvenile Sockeye Salmon Rearing Capacity of the Chignik Lakes Watershed
Source: PLoS One. 2026 May 19;21(5):e0349239. doi: 10.1371/journal.pone.0349239 (PMC13186349; doi:10.1371/journal.pone.0349239)
Supplement: S2 Table — (DOCX) [file pone.0349239.s002.docx]

**S2 Table. Linear regression statistics for maximum monthly surface water temperature.**

Ordinary least squares regressions of maximum monthly surface water temperature (°C; depths ≤ 10 m) on calendar year were fit separately for Black Lake and Chignik Lake in each of June, July, and August over the period 1990–2023.

| Lake | Month | *n*Years | Slope (°C yr^−1^) | SE | *t* | df | *p* | *R*^2^ | *r* |
| --- | --- | --- | --- | --- | --- | --- | --- | --- | --- |
| Black | June | 27 | 0.2230 | 0.0450 | 4.95 | 25 | 4.23 × 10−⁵ | 0.495 | 0.704 |
| Black | July | 25 | 0.2180 | 0.0405 | 5.37 | 23 | 1.85 × 10−⁵ | 0.557 | 0.746 |
| Black | Aug | 21 | 0.1790 | 0.0627 | 2.86 | 19 | 0.010 | 0.302 | 0.550 |
| Chignik | June | 30 | 0.1180 | 0.0330 | 3.58 | 28 | 0.001 | 0.313 | 0.560 |
| Chignik | July | 26 | 0.0813 | 0.0332 | 2.44 | 24 | 0.022 | 0.199 | 0.446 |
| Chignik | Aug | 21 | 0.0918 | 0.0517 | 1.77 | 19 | 0.092 | 0.142 | 0.377 |
