# Supplementary material for: Long-term changes in the Juvenile Sockeye Salmon Rearing Capacity of the Chignik Lakes Watershed
Source: PLoS One. 2026 May 19;21(5):e0349239. doi: 10.1371/journal.pone.0349239 (PMC13186349; doi:10.1371/journal.pone.0349239)
Supplement: Attachment — Submitted filename: Response_to_reviewers.docx [file pone.0349239.s005.docx]

Dear PLOS One Editorial Board,

We greatly appreciate the feedback provided by reviewers and have made the following revisions to our manuscript. Below we describe our responses to the comments provided by the reviewers and changes made to the manuscript.

In response to comments on our funding statement, we have removed funders from the acknowledgement section of the manuscript. A new funding statement can be found in the updated cover letter.

In response to the comment concerning the map in Figure 8 (now Figure 1), we have replaced the original figure with a newly generated map created entirely in R using publicly available spatial data. The shaded relief was independently generated in R from the USGS 3DEP DEM and therefore constitutes a new derived work. Hydrography data were obtained from the USGS National Hydrography Dataset, coastline data were obtained from NOAA, and the Alaska inset map was created using Natural Earth data.

Revisions made to reviewer comments by line number:

[47] Moved sentence to beginning of paragraph to begin Introduction with climate change > lake ecosystems > population responses

[63] Added citation (Quinn, 2018)

[96] Added sentence in figure 1 caption indicating 2018 stock collapse year and escapement goals.

[97] Added escapement goals to figure 1.

[101] Added “Comparing trends in rearing capacity between interconnected shallow (Black lake) and deep (Chignik lake) nursery lakes, we investigated asynchronous changes to habitat and population dynamics in a heterogeneous watershed.”

[104] Added corresponding to the typical 1-2 years of freshwater residency and 2-3 years of marine residency of sockeye salmon.” To clarify life cycle timing, and also provided relevant citations (Narvar 1966, Dhalberg 1979).

[249] Removed duplicate description of juvenile sockeye salmon and resident fish spp. CPUE calculation.

[288] Simplified results statement by removing mean summer temp results for Chignik lake. The increases were small, and complicated interpretation of key result that evidence for rapid temperature increases is lacking. Replaced with ; “Visual trends suggest mean monthly temperatures have increased minimally in both lakes since 1995, yet these trends were not statistically significant in Black (p > 0.05) or Chignik (p > 0.2) lake.”

[308] Figure 3 has now been uploaded.

[318] Moved figure reference to the end of the sentence and corrected capitalization.

[328] We believe Figure 5 should remain in the manuscript body as it highlights the increasing abundance (not just proportion) of Daphnia in Chignik lake, thereby providing additional evidence of improved growth opportunity for juvenile salmon in the ecosystem.

[340] Added ‘Non-metric Multidimensional scaling (NMDS)’

[341] Replaced ‘influence’ with ‘an association between atmospheric temperatures and community composition.’

[340] Added ‘Dashed vectors show months when temperature had no significant effect (p > 0.05) on species composition.’

[351] Added ‘,’ before ‘respectively’

[398] See line 509.

[419] Added; “These short term increases in water temperature can drive juvenile sockeye salmon to seek thermal refugia downstream in Chignik Lake (26).”

[431] This is supported by Carter & Schindler 2012 and Berger et al. 2010. I replaced ‘likely’ with ‘possible’.

[443] Clarified sentence.

[450] Edited paragraph for clarity

[502] We highlight the evidence that there was less predation on the zooplankton community in this lake, possibly indicating reduced recruitment of juvenile sockeye salmon into the lake. However, to quantify the relative effects of changing predation and climate variables on zooplankton community composition would require data that we do not have in our work. Thus, we have retained this statement as a likely explanation for the dynamics we observed in the zooplankton community but we are not able to further explore it in this paper.

[509] Moved paragraph proposing early life stage mortality event to discussion introduction.

[528] Added citation

[530] Added citation. Previous studies (Westley et al. 2008, Griffiths et al. 2013) in the watershed have identified earlier downstream emigration during warmer years.

[555] We agree that this statement is speculative and have reworded to clarify that changes in emigration patterns will obscure changes in stock-specific spawning success in each of the nursery lakes. Thus, we added the sentence. ‘Further, stock-specific spawning success would also obscure clear patterns in stock-specific growth patterns in Chignik Lake as density-dependent growth would be responsive to both intra-stock competition and inter-stock competition within the watershed.’

[563] Added citation

[611] We believe this discussion belong late in the Discussion because it is a hypothesis that is generated from the analyses presented above.

[629] Added “Our results highlight the importance of interconnected heterogeneous habitat networks in buffering long term populations. Further research on stock specific performance and juvenile life history strategies of Chignik sockeye salmon would help inform management strategies aimed at maintaining population diversity long term productivity of a mixed stock fishery.”

We thank the reviewers for their thoughtful and constructive feedback, which has improved the clarity of the manuscript. We hope that our revisions satisfactorily address all comments, and we welcome any further suggestions.

Sincerely,

Cirque Gammelin

On behalf of all authors
