# Supplementary material for: Long-term changes in the Juvenile Sockeye Salmon Rearing Capacity of the Chignik Lakes Watershed
Source: PLoS One. 2026 May 19;21(5):e0349239. doi: 10.1371/journal.pone.0349239 (PMC13186349; doi:10.1371/journal.pone.0349239)
Supplement: Attachment — Submitted filename: Response_to_reviewers_2.docx [file pone.0349239.s006.docx]

Dear PLOS One Editorial Board,

We greatly appreciate the feedback provided by reviewers and have made the following revisions to our manuscript. Below we describe our responses to the comments provided by the reviewers and changes made to the manuscript.

**Revisions made to reviewer comments, by line number:**

[68] & [83] fixed grammatical errors throughout

[Fig 1c] Fixed year 2020 on x-axis

[95] added paragraph to introduction addressing 2^nd^ conclusion comment. I intend this paragraph broaden background on other factors which may contribute to a stock collapse, and introduce alternate hypotheses earlier in the text.

‘With diverse and complex life histories, it can be challenging to identify and quantify the mechanisms behind sockeye salmon stock dynamics. Factors such as freshwater habitat quality, oceanic conditions, recruitment success, and harvest all contribute to the temporal stability of individual stocks [27,28]. While this study focuses on the hypothesized declines in freshwater rearing capacity in the Chignik watershed, it is possible multiple mechanisms contributed to the 2018 stock collapse. For example, an emerging hypothesis asks if extreme early winter flood events may have scoured sockeye salmon redds, resulting in poor egg and fry survival in the years preceding the collapse.’

[183] (previously 186) corrected temperature sampling year groups.

[301-325] – reported additional test statistics and edited results for clarity. Added S1 and S2 Tables [733]. Added sentence in methods [197],

[350] (previously 325) added “Species abbreviations for dominant zooplankton taxa are; *Daphnia* (Daph), calanoid copepod (Cal), cyclopoid copepod (Cyc), and *Bosmina* (Bos)” to Figure 6 caption.

[449] (previously 461) I can see what you mean. This paragraph intends to describe the relationship between temperature and sockeye bioenergetics in the watershed, how the life histories of both stocks reflect the thermal regimes of the two lakes, and to state the consistent and productive thermal conditions in both lakes.

I edited the entire ‘water temperature’ section of the discussion for clarity, removing duplicate statements and hopefully improved overall clarity. Notable changes below, see markup copy for all changes.

[435] removed sentence repeated in line 457

[448] added sentences describing the connection between freshwater life history strategies and lake specific thermal regimes.

[458] removed ‘Not only are smaller sockeye salmon at a competitive foraging disadvantage, they may also be less resilient to stressful thermal regimes.’

[467] added “While our data show average temperatures in the Chignik Lakes watershed are slowly rising, local climate and geomorphologic characteristics likely buffer against dramatic changes in water temperatures, and the thermal regimes of both lakes remain productive for juvenile sockeye salmon growth.”

[534] Rephrased paragraph to present observations as a possible explanation rather than evidence.

[565] (previously 585-557) added missing sentence

‘Further, stock-specific spawning success would also obscure clear patterns in stock-specific growth patterns in Chignik Lake as density-dependent growth would be responsive to both intra-stock competition and inter-stock competition within the watershed’

[659] added sentence addressing 1^st^ conclusion comment.

‘Although our analysis was conducted with comprehensive long term datasets, investigating numerous proxies for freshwater rearing capacity, it is possible that habitat features which support juvenile rearing underwent changes we were unable to measure with available data.’

[689] Response to 3^rd^ conclusion comment on management context

- The proposed management changes (described in the introduction) were revoked before this study concluded (line 89), and there have been no management changes since. The point of introducing the proposed management changes was to introduce the hypothesis of the study.
- I agree that it is necesary to acknowledge the importance of our results for informing future management, but only in the context of providing evidence of a resilient habitat network capable of supporting multiple productive sockeye salmon stocks and suggesting further research.
- See changes made in last paragraph of conclusion.

We thank the reviewers for their thoughtful and constructive feedback, which has improved the clarity of the manuscript. We hope that our revisions satisfactorily address all comments, and we welcome any further suggestions.

Sincerely,

Cirque Gammelin

On behalf of all authors
